# High Expression of Stem Cell-Related Genes in Polyps with Villous Features and High-Grade Dysplasia Support Malignant Phenotype and Colorectal Carcinogenesis

**DOI:** 10.31557/APJCP.2021.22.8.2429

**Published:** 2021-08

**Authors:** Ibrahim Sahin, Betül Gündoğdu, Ahmet Cevdet Ceylan, Haktan Bagis Erdem, Abdulgani Tatar

**Affiliations:** 1 *Department of Medical Genetics, University of Health Sciences, Dışkapı Yıldırım Beyazıt Training and Research Hospital, Ankara, Turkey. *; 2 *Department of Medical Genetics, Faculty of Medicine, Ataturk University, Erzurum, Turkey. *; 3 *Department of Medical Pathology, Faculty of Medicine, Ataturk University, Erzurum, Turkey. *; 4 *Department of Medical Genetics, Faculty of Medicine, Ankara Yildirim Beyazit University, Ankara, Turkey. *; 5 *Department of Medical Genetics, University of Health Sciences, Ankara Dr. Abdurrahman Yurtaslan Oncology Training and Research Hospital, Ankara, Turkey. *

**Keywords:** Colon cancer, tubular, villous, hyperplastic, OCT4, KLF4, SOX2, MYC, NANOG, REX1, CD133, LGR5

## Abstract

**Objective:**

The aim of this project is to identify the differences in expression levels of stem cell related genes (SCRGs) in normal colon tissue, histopathologically staged colon polyps and colon cancer, and to explain the role of SCRGs in the formation of CC and for contributing the practical usage of SCRGs in the diagnosis and treatment of CC.

**Methods:**

Normal colon tissue, hyperplastic polyps, histopathologically (HGD and LGD) staged tubular, tubulovillous and villous polyps and colon cancer paraffin tissue (FFPE) samples were used. Transcription factor genes (OCT4, KLF4, SOX2, MYC, NANOG, and REX1) and cell surface markers (CD133, LGR5), which are associated with embryonic stem cells, induced pluripotent stem cells, and cancer stem cells, have been selected for measuring expression levels from the selected tissues. After isolation of total RNA from FFPE tissues, SCRGs expressions were measured by RT-qPCR method.

**Results:**

SCRGs expression differences were detected in normal–adenoma–cancer progression. A significant increase was observed in the expression of LGR5 (p: 0.01) and PROM1 (p: 0.005) genes in villous HGD polyps, LGR5 (p: 0.003) gene in G1, and LGR5 (p: 0.0002) and MYC (p: 0.002) genes in G2 stage tumor tissues. When compared with each other, a significant increase in SCRGs expression is noticeable in the formation from adenoma to cancer tissues regarding malign phenotype.

**Conclusion:**

This study shows that the increase of SCRGs expressions occurs with high-grade dysplasia (HGD), villous features, and the malignant phenotype. Increased expression levels of LGR5, PROM1, KLF4, SOX2, and MYC in HGD and cancerous tissues support the malignant phenotype and the existence of cancer stem cells and demonstrate that they can be used to assess diagnosis and prognosis. Identification of tissue-specific SCRGs expressions will help design new therapies to control the development and progression of colonic neoplasia.

## Introduction

Colorectal cancer (CC) is one of the most common cancers worldwide, and between one and two million new cases are diagnosed each year, making CC the fourth most common cancer. It is the third most common cause of cancer death, with 700,000 deaths annually. Although one of the most known cancers, the reality that CC causes over 2 million new cases, and over 1 million deaths annually shows that this problem should be better enlightened (Rawla et al., 2019). 

From benign adenoma to malignant adenocarcinoma and metastasis, CC formation follows a long, gradual process (Harris and McCormick, 2010). All types of adenomas show dysplasia with abnormal glandular architecture and damaged intracellular structures. The degree of dysplasia is varied, and in severe dysplasia, malignancy risk increases. 

Stem cells (SCs) can be divided into three groups: embryonic, germinal, and progenitor somatic SCs (Reya et al., 2001). About 25 transcription factors (TFs) are expressed in stem cells. OCT4, SOX2, KLF4, and NANOG form an essential regulatory network for the maintenance and self-renewal of embryonic stem cells. These TFs are strongly expressed in embryonic stem cells, but are mostly suppressed in normal somatic cells, except for a few adult stem cell types. Increasing evidence suggests that embryonic specific TFs are overexpressed in human tumor tissues, implying the existence of cancer stem cells. CSCs are distinguished by the expression of stemness-related markers seen in embryonic stem cells (ESCs) and adult stem cells, the two major kinds of human stem cells. Retrospective investigations on patient cohorts have also linked TF expression to survival outcomes in certain tumor types, suggesting that TF expression levels may help predict patient prognosis. Detecting the amount of expression of these TFs may therefore help in tumor diagnosis, classification, and treatment options (Zhao et al., 2017).

Tumors have a heterogeneous structure consisting of multiple structures and functions of a mixture of cells. Stem cells are potential candidates for malignant transformation due to their capacity to self-renew and dedifferentiate, resulting in the acquisition of both genetic and epigenetic changes needed for carcinogenesis (Wuputra et al., 2020). Cancer stem cells (CSCs) are known to lie at the center of this heterogeneous structure (Ghoneim et al., 2020). Cancer stem cells (CSCs), with their capacity to self-renew and differentiate into several lineages, are self-renewing cells responsible for tumor onset, recurrence, resistance to treatment, differentiation, invasion, and metastasis (Nguyen et al., 2012). 

OCT4, SOX2, NANOG, and KLF4, which are pluripotency factors specific to embryonic SCs, are called induced pluripotent stem cells (iPSC) or Yamanaka factors. These factors, which have been identified in embryonic stem cells, were first used by Yamanaka et al. (2006) and then by Wernig et al. (2007) in the transformation of adult cells into pluripotent cells (Takahashi and Yamanaka, 2006; Wernig et al., 2007). OCT4, NANOG, SOX2, and REX1 are the four fundamental stemness transcriptional factors responsible for cellular pluripotency and differentiation (Javed et al., 2021). ESC regulation, cellular reprogramming, and carcinogenesis may all be regulated by the same fundamental master regulators. Treatment resistance of lethal tumors was linked to OCT4, SOX2, and NANOG in cancer patients (Hepburn et al., 2019).

It has been reported that cells that initiate colon cancer express markers such as LGR5 and CD133 (Srinivasan et al., 2016). LGR5 is a G-protein coupled receptor that may interact with and activate Wnt signaling by binding to the furin-like repeat FU2 domain of R-spondin (de Lau et al., 2014). LGR5 has been identified as a potential marker for CC stem cells. LGR5 stimulation of Wnt and TGF-b signaling in cancer stem cells has just recently been discovered. LGR5 overexpression promotes treatment resistance and cancer stemness in both brain tumors and CC. The presence of LGR5 enhances cell-cell adhesion, which promotes stemness and inhibits invasiveness and migration (Javed et al., 2021). These results point to LGR5 as a possible marker of CC stem cells.

MYC has a gene activation function, a finding consistent with MYC’s ability to regulate multiple coactivator complexes (Adhikary and Eilers, 2005). Oncogenic deregulation of MYC expression produces cells with tumor phenotype during normal development. REX1 (ZFP42 or REXO1) is a known marker of pluripotency and is usually found in undifferentiated embryonic stem cells and teratocarcinoma cells. Besides being a pluripotent marker, its regulation is also critical to maintaining the pluripotent state (Filipczyk et al., 2015). 

In this study, we performed an expression study on 116 patients to explain the importance of stem cell related genes (SCRGs) in normal colon tissue, histopathologically staged colon polyps and colon cancer (CC), and the role of SCRGs in the formation of CC and for contributing the practical usage of SCRGs in the diagnosis and treatment of CC. Our data broadens the knowledge of SCRGs and provides insights for colon carcinogenesis. 

## Materials and Methods

*Tumor Samples and Patients*

Formalin-fixed paraffin-embedded (FFPE) tissue sample blocks were taken from Ataturk University Faculty of Medicine, Department of Pathology. Healthy colon tissue of CC patients was used as normal control. Normal colon tissue, hyperplastic polyps, histopathologically (HGD and LGD) staged tubular, tubulovillous and villous polyps, and colon cancer FFPE samples were used (Table 1). Except for the control group, ten groups were formed (Table 1). Each group was compared with the control group, a fold change of 2 or more than 2 was considered significant.

*RNA Extraction, cDNA synthesis, and qRT-PCR*

MiRNeasy FFPE Kit (Qiagen, Germany) was used for isolation with QIAcube (Qiagen, Germany). Total RNA was isolated according to the manufacturer’s protocol. RNA quantity and quality were measured with QIAxpert (Qiagen, Germany). A260: A230 ratio was greater than 1,7, and A260: A280 ratio was between 1,8 to 2. Preamplification step was performed with RT2 PreAMP cDNA Synthesis Kit (Qiagen, Germany) on samples containing sufficient quantity and quality of RNA. PreAmp primers were specially designed and used for the POU5F1 (OCT4), KLF4, SOX2, MYC, NANOG, REX1, PROM1 (CD133), LGR5, and ACTB genes. This step was added due to the amount and purity of RNA obtained from FFPE tissues could be variable and low. Steps were followed according to the manufacturer’s protocol, and the quantity and quality of RNA were increased. 100 ng of RNA was used for cDNA synthesis.

An expression study was performed using RT2 qPCR Primer Assay (Qiagen, Germany) and SYBR Green dye. RNAfold and Beacon Primer Designer (Premier Biosoft International) software were used to evaluate the structure and functionality of the primers. Real-time PCR protocol for Rotor-Gen Q (Qiagen, Germany) and RT2 qPCR Primer Assay (Qiagen, Germany) was applied according to the manufacturer’s protocol. To check the PCR specificity, dissociation (melting) curve analysis was carried out. In a melting curve program using real-time loop software, the first derivative dissociation curve was constructed for each well. A single peak was seen for each gene in each reaction.

*Data Analyses*

The data were evaluated in the SPSS version 20.0 statistics program. Student T-test was used to compare the two independent groups in terms of numerical variables with normal distribution, and p <0.05 was considered statistically significant. Graphics were prepared with Python (version 3.9.2).

## Results

The mean age of the enrolled patients was 61 years. SCRGs expressions were present in normal tissues, possibly due to intestinal stem cells; even their expression levels were higher than most polyps. In hyperplastic polyps (group 1), there was a significant decrease in the expression of PROM1 (p: 0.01), POU5F1 (p: 0.02), LGR5 (p: 0.01) and REX1 (p: 0.01) genes compared to the control group. In tubular LGD polyps (group 2), a significant decrease was found in the expressions of POU5F1 (p: 0.04) and REX1 (p: 0.02) genes compared to the control group. In tubular HGD polyps (group 3), a significant decrease was observed in the expressions of POU5F1 (p: 0.03) and PROM1 (p: 0.047) genes compared to the control group. Similarly, in tubulovillous LGD polyps (group 4), a significant decrease in the expression of POU5F1 (p: 0.03) and PROM1 (p: 0.046) genes was observed compared to the control group. There was no statistically significant difference in tubulovillous HGD (group 5) and villous LGD polyps (group 6) compared to the control group (Table 2). 

In villous HGD polyps (group 7), a significant increase was observed in the expression of LGR5 (p: 0.01) and PROM1 (p: 0.005) genes compared to the control group. A significant increase in expression of LGR5 (p: 0.003) gene was found in G1 stage tumor tissues (group 8) compared to the control group. A significant increase was observed in the expression of LGR5 (p: 0.0002) and MYC (p: 0.002) genes in G2 stage tumor tissues (group 9) compared to the control group. There was no statistically significant difference in G3 stage tumor tissues (group 10) compared to the control group. Although some genes in some groups show more than two-fold change expressions, this change seems statistically insignificant due to the large difference between the highest and lowest Delta Ct values (Table 2).

**Figure 1:**
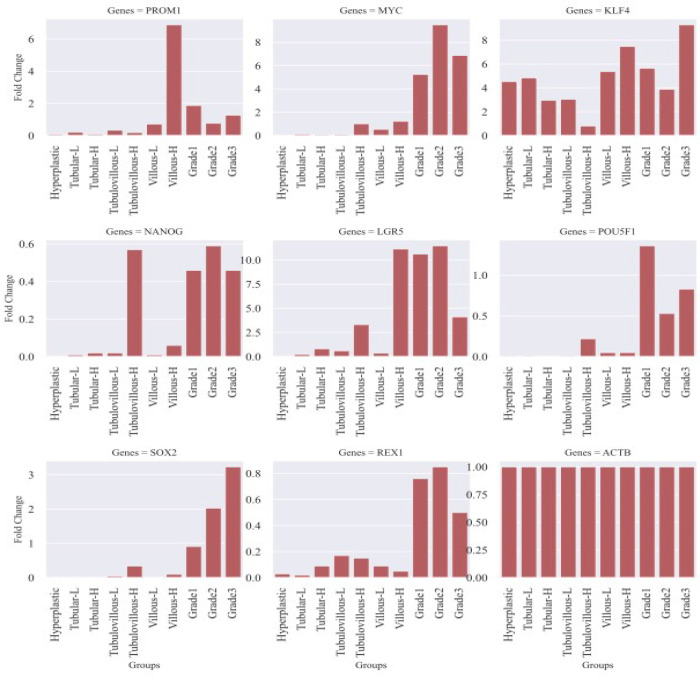
Log2 Fold Change of the Stem Cell-Related Genes in Selected Tissues

**Figure 2:**
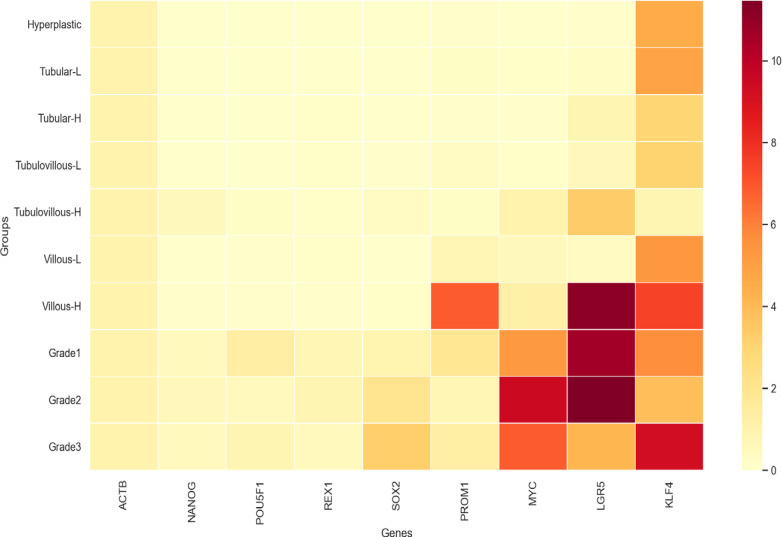
Heatmap: Showing Increased Expression Levels of the Stem Cell-Related Genes from benign to Malignant Phenotype

When compared with each other, a significant increase in SCRGs expression is noticeable in the formation from adenoma to cancer tissues regarding malign phenotype. Our study supports that cells with stem cell-like features increase from adenoma to cancer (Figures 1 and 2). 

**Table 1:** Number of Groups and Histopathologically Staged Tissue Types Used in the Study

Groups	Tissue types	Number (n)
Control	Normal	18
Group 1	Hyperplastic	12
Group 2	Tubular-L	9
Group 3	Tubular-H	9
Group 4	Tubulovillous-L	10
Group 5	Tubulovillous -H	9
Group 6	Villous-L	9
Group 7	Villous-H	4
Group 8	Cancer-G1	10
Group 9	Cancer -G2	12
Group 10	Cancer -G3	14
	Total	116

**Table 2:** Comparison of Gene Expression Fold Changes of the Groups Compared to the Control Group

	PROM1	MYC	KLF4	NANOG	LGR5	POU5F1	SOX2	REX1	ACTB
Hyperplastic (Group 1)									
Fold Change	0.07	0.03	4.53	0.00	0.06	0.01	0.00	0.03	1.00
* p*-value	0.01	0.15	0.57	0.17	0.01	0.02	0.19	0.01	0.00
Tubular L (Group 2)									
Fold Change	0.21	0.11	4.84	0.01	0.23	0.01	0.00	0.02	1.00
* p*-value	0.0503	0.22	0.77	0.25	0.17	0.04	0.22	0.02	0.00
Tubular-H (Group 3)									
Fold Change	0.08	0.08	2.96	0.02	0.82	0.00	0.00	0.09	1.00
* p*-value	0.047	0.25	0.64	0.24	0.24	0.03	0.26	0.13	0.00
Tubulovillous-L (Group 4)									
Fold Change	0.34	0.09	3.05	0.02	0.61	0.01	0.05	0.17	1.00
* p*-value	0.046	0.21	0.36	0.22	0.15	0.03	0.24	0.77	0.00
Tubulovillous -H (Group 5)									
Fold Change	0.19	1.02	0.81	0.57	3.32	0.22	0.34	0.15	1.00
* p*-value	0.09	0.57	0.91	0.68	0.19	0.27	0.49	0.15	0.00
Villous-L (Group 6)									
Fold Change	0.72	0.54	5.37	0.01	0.37	0.05	0.00	0.09	1.00
* p*-value	0.48	0.59	0.23	0.26	0.41	0.08	0.26	0.06	0.00
Villous -H (Group 7)									
Fold Change	6.88	1.23	7.48	0.06	11.14	0.05	0.10	0.05	1.00
* p*-value	0.005	0.62	0.98	0.44	0.01	0.19	0.51	0.11	0.00
Cancer-G1 (Group 8)									
Fold Change	1.87	5.24	5.65	0.46	10.62	1.36	0.91	0.76	1.00
* p*-value	0.13	0.15	0.08	0.33	0.003	0.36	0.87	0.52	0.00
Cancer -G2 (Group 9)									
Fold Change	0.77	9.49	3.89	0.59	11.46	0.53	2.03	0.85	1.00
* p*-value	0.36	0.002	0.60	0.26	0.0002	0.10	0.81	0.73	0.00
Cancer -G3 (Group 10)									
Fold Change	1.27	6.88	9.28	0.46	4.12	0.83	3.23	0.50	1.00
* p*-value	0.23	0.11	0.12	0.55	0.14	0.87	0.055	0.26	0.00

## Discussion

The annual incidence of colon cancer is increasing. Colon cancer is considered to be caused mainly by adenomatous polyps. The transformation of a polyp into invasive carcinoma is related to its histopathological type and size; the degree of dysplasia also determines the transformation of adenoma into cancer. High-grade dysplasia (HGD) has a higher risk of transforming into cancer than low-grade dysplasia (LGD). Neoplastic cells penetrating the basement membrane and acquiring metastatic properties mark a critical point in the progression from adenoma to carcinoma (Ahnen, 2011). 

Tumors have a heterogeneous structure, with cancer stem cells (CSCs) at the center of the structure (Ghoneim et al., 2020). In many cancers, CSCs are self-regenerative cells responsible for tumor onset, recurrence, resistance to treatment, differentiation, invasion, and metastasis (Hepburn et al., 2019). Although they are rare in cancer tissue, they are seen as one of the most significant causes of cancer treatment failure (Clevers, 2011; Nguyen et al., 2012). DNA methylation studies have revealed that CSCs resemble embryonic stem cells rather than normal stem cells (Wong et al., 2008). Therefore, in our study, we selected transcription factor genes (OCT4, KLF4, SOX2, MYC, NANOG, and REX1) and cell surface markers (CD133, LGR5) associated with embryonic stem cells, induced pluripotent stem cells, and CSCs. Our research findings support the increase in the number of stem cell-like cells in the progression from adenoma to cancer.

We saw an increase in POU5F1 expression in tissue with villous and high-grade dysplasia compared to other polyp tissue, but this increase was not statistically significant compared to normal tissue. In their study on metastasis in colon cancer, Singovski et al. showed that the metastatic features of cancer reprogrammed with OCT4, SOX2, KLF4, and +/- cMYC increased. NANOG expression also increased, but LGR5 decreased (Singovski et al., 2016). OCT4 expression in the intestine has been reported to cause dysplasia by inhibiting cellular differentiation, as in ES cells (Yamada and Watanabe, 2010).

Although the expression of KLF4 detected in tissue with villous and high-grade dysplasia is higher than that of other polyp tissue, it is also highly expressed in normal colonic tissue. Hu et al. (2011) stated that KLF4 expression is increased in normal–adenoma–cancer progression (Hu et al., 2011). 

MYC expression, which is low in hyperplastic and tubular polyps, increases with the acquisition of HGD and villous features and peaks in cancerous tissues. Oncogenic deregulation of MYC expression produces cells with the tumor phenotype during normal development. High MYC expression levels can block cell differentiation and improve the self-renewal of processed and differentiated cells. During tumor progression, MYC promotes the formation of cancer-initiating cells that retain developmental plasticity 0(Eilers and Eisenman, 2008; Wuputra et al., 2020).

In colon cancer, increased levels of NANOG expression are associated with advanced cancer stages and a poor prognosis. NANOG expression is likely functionally crucial in colon cancer, as overexpression of the NANOG gene increases the proliferation of cancer cells (Meng et al., 2010). As the cells begin to differentiate, REX1 becomes stiff and is abruptly downregulated (Wang et al., 2006). In our study, REX1 expression levels in polyp and cancer tissue were low compared to levels in normal tissue. However, REX1 expression increases in the progression from adenoma to cancer. It has been reported that the REX1 gene product is a direct target of the NANOG gene; REX1 expression is increased by SOX2 and OCT-3/4 (Shi et al., 2006).

The increased expression of the CD133 gene, especially in villous HGD polyps (group 7), supports the presence of cells that highly express CD133. LGR5 is one of the most important markers of colon CSCs (Langan et al., 2013; Javed et al., 2021). Its expression gradually increases, especially in polyps showing high-grade dysplasia (groups 3, 5, and 7) and cancer tissues. The increasing expression pattern from normal tissue to cancer is a good indicator that the dominance of cells expressing LGR5 in the environment has increased. The distribution of the LGR5 gene expression increase in adenomas indicates that a stem cell/progenitor cell hierarchy is preserved in early neoplastic lesions (Barker et al., 2009).

The most relevant cancer formation pathways, such as Wnt, BMP4, and TGFb, are associated with SCRGs. These signal networks are responsible for tissue homeostasis. The Wnt pathway, which is closely related to the oncogenic phenotype, plays an essential role in the growth and control of stem cell functions in cells. Ninety percent of colorectal cancers produce β-catenin and APC mutations responsible for Wnt pathway activation (Vermeulen et al., 2010; Javed et al., 2021).

The most significant finding here is the predominance of LGR5, PROM1, MYC, and KLF4 in villous HGD polyps. LGR5 and CD133 are the most important colon CSC markers. Many studies show that LGR5 shows tumor-initiating cells in the intestinal system (Asfaha et al., 2015). Similarly, cells expressing CD133 have been implicated in the onset of cancer (Curley et al., 2009). This research shows that CSC-related factors occur with HGD and villous features, supporting the hypothesis that polyps with villous features and HGD have a marked tendency to develop cancer. LGR5, PROM1, and MYC expression levels can be helpful in diagnosis and prognosis.

In conclusion, changes in the expression levels of the chosen genes in cancer tissues are noteworthy. This supports the hypothesis that stem cell-related factors use common pathways to perform their functions and are interdependent. While some comparisons are statistically insignificant due to high SCRGs expressions in normal tissues, likely in relation to intestinal stem cells, it is noteworthy that SCRGs show an increasing pattern of expression from adenoma to cancer. Identification of tissue-specific SCRGs expressions will aid in the design of new treatments for controlling the development and progression of colonic neoplasia. The results obtained in this study may form the basis of new-generation genetic screening tests. Similarly, the development and application of new and sensitive specific biomarkers in the near future will aid in developing diagnostic strategies and improving prognoses.

## Author Contribution Statement

IS designed the research, analyzed, and interpreted the genetic results (including the coding part with Python version 3.9.2), wrote the manuscript, and approved the final manuscript. BG collected the FFPEs, performed the histopathological tissue analysis, reviewed the manuscript, and approved the final manuscript. ACC collected the data, analyzed, and interpreted the genetic results, reviewed the manuscript, and approved the final manuscript. HBE collected the data, analyzed, and interpreted the genetic results, reviewed the manuscript, and approved the final manuscript. AT collected the data, analyzed, and interpreted the genetic results, reviewed the manuscript, and approved the final manuscript.

## Availability of code and data

The data and the code of the current study are available from the corresponding author on reasonable request.

## References

[B1] Adhikary S, Eilers M (2005). Transcriptional regulation and transformation by Myc proteins. Nat Rev Mol Cell Biol.

[B2] Ahnen DJ (2011). The American College of gastroenterology emily couric lecture the adenoma-carcinoma sequence revisited: Has the era of genetic tailoring finally arrived. Am J Gastroenterol.

[B3] Asfaha S, Hayakawa Y, Muley A (2015). Krt19+/Lgr5- cells are radioresistant cancer-initiating stem cells in the colon and intestine. Cell Stem Cell.

[B4] Barker N, Ridgway RA, Van Es JH (2009). Crypt stem cells as the cells-of-origin of intestinal cancer. Nature.

[B5] Clevers H (2011). The cancer stem cell: Premises, promises and challenges. Nat Med.

[B6] Curley MD, Therrien VA, Cummings CL (2009). CD133 expression defines a tumor initiating cell population in primary human ovarian cancer. Stem Cells.

[B7] Eilers M, Eisenman RN (2008). Myc’s broad reach. Genes Develop.

[B8] Filipczyk A, Marr C, Hastreiter S (2015). Network plasticity of pluripotency transcription factors in embryonic stem cells. Nat Cell Biol.

[B10] Harris TJR, McCormick F (2010). The molecular pathology of cancer. Nat Rev Clin Oncol.

[B11] Hepburn AC, Steele RE, Veeratterapillay R (2019). The induction of core pluripotency master regulators in cancers defines poor clinical outcomes and treatment resistance. Oncogene.

[B12] Hu R, Zuo Y, Zuo L (2011). KLF4 expression correlates with the degree of differentiation in colorectal cancer. Gut Liver.

[B13] Javed Z, Javed Iqbal M, Rasheed A (2021). Regulation of hedgehog signaling by miRNAs and nanoformulations: A Possible Therapeutic Solution for Colorectal Cancer. Front Oncol.

[B14] Langan RC, Mullinax JE, Raiji MT (2013). Colorectal cancer biomarkers and the potential role of cancer stem cells. J Cancer.

[B15] de Lau W, Peng WC, Gros P, Clevers H (2014). The R-spondin/Lgr5/Rnf43 module: Regulator of Wnt signal strength. Genes Develop.

[B16] Meng HM, Zheng P, Wang XY (2010). Overexpression of nanog predicts tumor progression and poor prognosis in colorectal cancer. Cancer Biol Ther.

[B17] Nguyen LV, Vanner R, Dirks P, Eaves CJ (2012). Cancer stem cells: An evolving concept. Nat Rev Cancer.

[B18] Rawla P, Sunkara T, Barsouk A (2019). Epidemiology of colorectal cancer: Incidence, mortality, survival, and risk factors. Przeglad Gastroenterol.

[B19] Reya T, Morrison SJ, Clarke MF, Weissman IL (2001). Stem cells, cancer, and cancer stem cells. Nature.

[B20] Shi W, Wang H, Pan G (2006). Regulation of the Pluripotency Marker Rex-1 by Nanog and Sox2. J Biol Chem.

[B21] Singovski G, Bernal C, Kuciak M (2016). In vivo epigenetic reprogramming of primary human colon cancer cells enhances metastases. J Mol Cell Biol.

[B22] Srinivasan T, Walters J, Bu P (2016). NOTCH signaling regulates asymmetric cell fate of fast- and slow-cycling colon cancer-initiating cells. Cancer Res.

[B23] Takahashi K, Yamanaka S (2006). Induction of pluripotent stem cells from Mouse Embryonic and Adult Fibroblast Cultures by Defined Factors. Cell.

[B24] Vermeulen L, De Sousa E Melo F, Van Der Heijden M (2010). Wnt activity defines colon cancer stem cells and is regulated by the microenvironment. Nat Cell Biol.

[B25] Wang J, Rao S, Chu J (2006). A protein interaction network for pluripotency of embryonic stem cells. Nature.

[B26] Wernig M, Meissner A, Foreman R (2007). In vitro reprogramming of fibroblasts into a pluripotent ES-cell-like state. Nature.

[B27] Wong DJ, Liu H, Ridky TW (2008). Module map of stem cell genes guides creation of epithelial cancer stem cells. Cell Stem Cell.

[B28] Wuputra K, Ku CC, Wu DC (2020). Prevention of tumor risk associated with the reprogramming of human pluripotent stem cells. J Exp Clin Cancer Res.

[B29] Yamada Y, Watanabe A (2010). Epigenetic codes in stem cells and cancer stem cells. Adv Genet.

[B30] Zhao W, Li Y, Zhang X (2017). Stemness-related markers in cancer. Cancer Transl Med.

